# A trait‐based framework for stream algal communities

**DOI:** 10.1002/ece3.1822

**Published:** 2015-12-08

**Authors:** Katharina Lange, Colin Richard Townsend, Christoph David Matthaei

**Affiliations:** ^1^ Department of ZoologyUniversity of OtagoPO Box 56DunedinNew Zealand

**Keywords:** Agricultural land use, benthic stream algae, conceptual model, multiple stressors, periphyton

## Abstract

The use of trait‐based approaches to detect effects of land use and climate change on terrestrial plant and aquatic phytoplankton communities is increasing, but such a framework is still needed for benthic stream algae. Here we present a conceptual framework of morphological, physiological, behavioural and life‐history traits relating to resource acquisition and resistance to disturbance. We tested this approach by assessing the relationships between multiple anthropogenic stressors and algal traits at 43 stream sites. Our “natural experiment” was conducted along gradients of agricultural land‐use intensity (0–95% of the catchment in high‐producing pasture) and hydrological alteration (0–92% streamflow reduction resulting from water abstraction for irrigation) as well as related physicochemical variables (total nitrogen concentration and deposited fine sediment). Strategic choice of study sites meant that agricultural intensity and hydrological alteration were uncorrelated. We studied the relationships of seven traits (with 23 trait categories) to our environmental predictor variables using general linear models and an information‐theoretic model‐selection approach. Life form, nitrogen fixation and spore formation were key traits that showed the strongest relationships with environmental stressors. Overall, FI (farming intensity) exerted stronger effects on algal communities than hydrological alteration. The large‐bodied, non‐attached, filamentous algae that dominated under high farming intensities have limited dispersal abilities but may cope with unfavourable conditions through the formation of spores. Antagonistic interactions between FI and flow reduction were observed for some trait variables, whereas no interactions occurred for nitrogen concentration and fine sediment. Our conceptual framework was well supported by tests of ten specific hypotheses predicting effects of resource supply and disturbance on algal traits. Our study also shows that investigating a fairly comprehensive set of traits can help shed light on the drivers of algal community composition in situations where multiple stressors are operating. Further, to understand non‐linear and non‐additive effects of such drivers, communities need to be studied along multiple gradients of natural variation or anthropogenic stressors.

## Introduction

The distribution and abundance of a species is related to its ability, conferred by a particular set of biological traits, to withstand habitat filters acting at scales ranging from climatic region to landscapes and microhabitats (Grime 1979; Tilman 1982; Townsend and Hildrew 1994; Poff 1997). The study of species traits can provide insights into the mechanisms driving community and ecosystem processes along gradients of influential variables, including responses to anthropogenic change. Thus, scientists have shown increasing interest in developing trait‐based approaches in freshwater (Biggs et al. 1998b; Reynolds et al. 2002; Weithoff 2003; Burliga et al. 2004; Litchman and Klausmeier 2008; Lange et al. 2011), marine (Alves‐de‐Souza et al. 2008; Litchman and Klausmeier 2008; Barton et al. 2013; Edwards et al. 2013) and terrestrial ecosystems (De Deyn et al. 2008; Díaz et al. 2013; Marteinsdóttir and Eriksson 2014). The investigated traits are usually (1) mechanistically related to evolutionary processes, (2) founded on abiotic rather than biotic factors (Poff 1997), (3) easily measurable and (4) reflective of the main processes affecting organism performance (Weithoff 2003; Kruk et al. 2010). Despite the wealth of information about individual taxa in the literature, the assembly of information for morphological, physiological, behavioural and life‐history traits for entire communities remains a substantial challenge (Litchman and Klausmeier 2008).

The trait‐based approach has a long history for terrestrial plants (Grime 1979; Tilman 1980), but has only recently been considered for freshwater periphyton (e.g. Biggs et al. 1998b). Freshwater periphyton contains a diverse, polyphyletic assembly of photosynthetically active protists and cyanobacteria that play crucial roles in fluxes of matter and energy, especially in lotic and shallow lentic environments. (Note that, for simplicity, we henceforth use the term “algae” to describe this community.) Traits such as pigment composition, motility and nitrogen fixation contribute to regulating a species’ ability to acquire light and nutrients, whereas life forms, mode of attachment and life‐history traits help determine their ability to withstand and recover from physical disturbances and grazing (Biggs et al. 1998b; Litchman and Klausmeier 2008). The conceptual framework with four trait classes (morphological, physiological, behavioural and life‐history) that forms the basis of our study is shown in Table 1. This framework was adapted for benthic stream algae from the trait‐based concept proposed by Litchman and Klausmeier (2008) for marine and freshwater phytoplankton communities. It also draws on the “habitat matrix conceptual model for stream periphyton” developed by Biggs et al. (1998b).

**Table 1:** Conceptual framework of morphological, physiological, behavioural and life‐history traits of stream algae and cyanobacteria in relation to resource acquisition and resistance to disturbance (adapted in part from Litchman and Klausmeier 2008 and Biggs et al. 1998b). Disturbance is defined as any discrete event including severe low flows or stream drying, but also extreme fluctuations in temperature and oxygen conditions (which may arise due to intensive agriculture; Allan 2004) or grazing by herbivorous animals

	Resource acquisition (nutrients and light)	Resistance and resilience to disturbance (physicochemical extremes, flow, grazing)
Morphology	**Cell size**	**Cell size**
	MR1: Smaller cells have higher nutrient uptake rates relative to larger cells (advantage under nutrient‐limiting conditions)^1,2,3^	MD1: Smaller cells exhibit higher growth rates and resilience (more common under high disturbance regimes)^2,3^
	**Life form**	**Life form**
	MR2: Filamentous forms project above the biofilm reaching into the water column (advantage in resource gathering)^2,5^	MD2: Filamentous forms are more susceptible to drag by high shear stress (less common under high disturbance regimes)^2,6^
		**Attachment to substratum**
		MD3: Algae with stronger attachment to the substratum are more likely to remain attached to surfaces under conditions of high shear stress^2,6^
Physiology	**Nitrogen fixation**	
	PR1: N‐fixing algae access an additional nitrogen source (advantage under nutrient‐limiting conditions)^1,2^	
Behaviour	**Motility**	**Motility**
	BR1: Actively motile organisms can move to the optimal layers within the biofilm matrix (advantage in resource gathering)^4,5^	BD1: Actively motile organisms can avoid burial by siltation (advantage under low‐flow depositional conditions)^7^
Life‐history		**Main reproductive technique**
		LHD1: Fission generally produces smaller propagules than fragmentation (advantageous for dispersal and recolonization after disturbance)^2^
		**Spore formation**
		LHD2: Formation of thick‐walled, dormant spores (akinetes, oospores, zygospores) enables cells to endure unfavourable conditions^1,8^

Our specific hypotheses in the four different trait classes are abbreviated as follows: MR1‐MR2, morphology and resources; MD1–MD3, morphology and disturbance; PR1, physiology and resources; BR1, behaviour and resources; BD1, behaviour and disturbance; LHD1–LHD2, life‐history and disturbance.

Hypotheses are based on (1) Litchman and Klausmeier (2008) (2) Biggs et al. (1998b) (3) Passy (2007b), (4) Weithoff (2003), (5) Passy (2007a); (6) Steinman (1996), (7) Wagenhoff et al. (2013) and (8) Agrawal (2009).

Agricultural land use and flow regime alterations are two key drivers of change in stream ecosystems, affecting resource supply, physicochemical habitat characteristics and matter and energy cycling. Agricultural intensification can impose a variety of stressors in streams, including nutrient enrichment and augmented fine sediment inputs, increased frequency and intensity of disturbances via erosional events, nutrient spikes, temperature extremes, lowered base flows and more flashy flow patterns (Allan 2004). Water abstraction, by reducing stream discharge and current velocities downstream, may increase the frequency and/or severity of low‐flow events with associated depositional conditions, enhancing the accumulation of fine sediment and organic matter, and may also cause the occasional extreme stream drying event (Lake 2003; Dewson et al. 2007). This variety of consequences for stream environments makes it difficult to tease apart the individual and combined effects of the reach‐scale stressors involved (e.g. Biggs et al. 1998b; Wagenhoff et al. 2013; Piggott et al. 2015), not least because, due to interactions, combined stressor effects can be larger or smaller than expected based on knowledge of the effects of individual stressors (Folt et al. 1999). Nevertheless, ecologists may gain insights into mechanisms by which agricultural land use and flow reduction act on stream communities by examining their biological traits (Kelly 2013).

To our knowledge, no studies have investigated the combined effects of multiple, simultaneously operating agricultural stressors on a comprehensive set of stream periphyton traits. Most previous studies have considered only a few traits and were conducted in mesocosms (Magbanua et al. 2013; Wagenhoff et al. 2013; Piggott et al. 2015) while field studies are scarce (but see Passy 2007a; Berthon et al. 2011; Law et al. 2014). Thus, we designed a “natural experiment” to investigate the relationships of seven traits (with 23 trait categories) of stream algae along pre‐defined gradients of agricultural land use and hydrological alteration at multiple sites in a single river catchment. In particular we wished to (1) examine the applicability of our conceptual model by testing the ten specific hypotheses shown in Table 1, (2) determine which traits showed the strongest relationships with stressor gradients, and (3) investigate whether these traits can help unravel the mechanisms that drive the development of algal communities exposed to multiple stressors.

## Methods

### Study sites

We selected 43 sites in the Manuherikia River catchment in Central Otago, South Island of New Zealand, one of the driest catchments in the country. About half its area has been converted from native tussock grasslands to high‐intensity sheep/beef farming, especially at lower elevations (Lange et al. 2014a). Our measure of FI (farming intensity) was the percentage of each sub‐catchment in the category “high‐producing exotic grassland” (spatial distribution of land cover types available from the Land Cover Database II, Ministry for the Environment 2008, and detailed delineation of stream reaches and sub‐catchments from the River Environmental Classification, Ministry for the Environment 2010). The % FI index ranged from 0 to 95% in the studied sub‐catchments. Because data on the amount of water diverted from each stream site were not available, we used modelled streamflows for the Manuherikia catchment under different land‐use scenarios to estimate WA (water abstraction) intensity at each site (Lange et al. 2014a). These flows were simulated by Kienzle and Schmidt (2008) using the ACRU model (Agricultural Catchments Research Unit; University of Natal, South Africa). For each sub‐catchment, % WA was calculated as the percentage of streamflow reduction from the *Dryland Scenario* (no water abstracted) to the *Current Scenario*. This index (range 0–92% in the studied sub‐catchments) was based on mean streamflow across five irrigation seasons (1 October–31 April) from 1999/2000 to 2004/2005.

**Figure 1:**
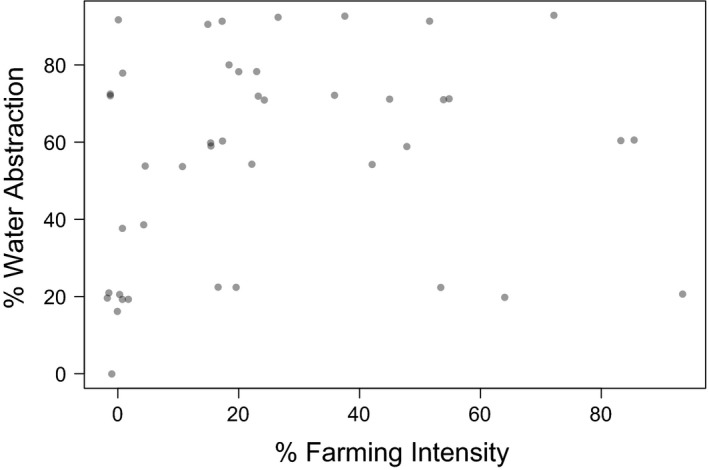
Distribution of the 43 stream sites along the gradients of % Farming Intensity and % Water Abstraction.

**Figure 2:**
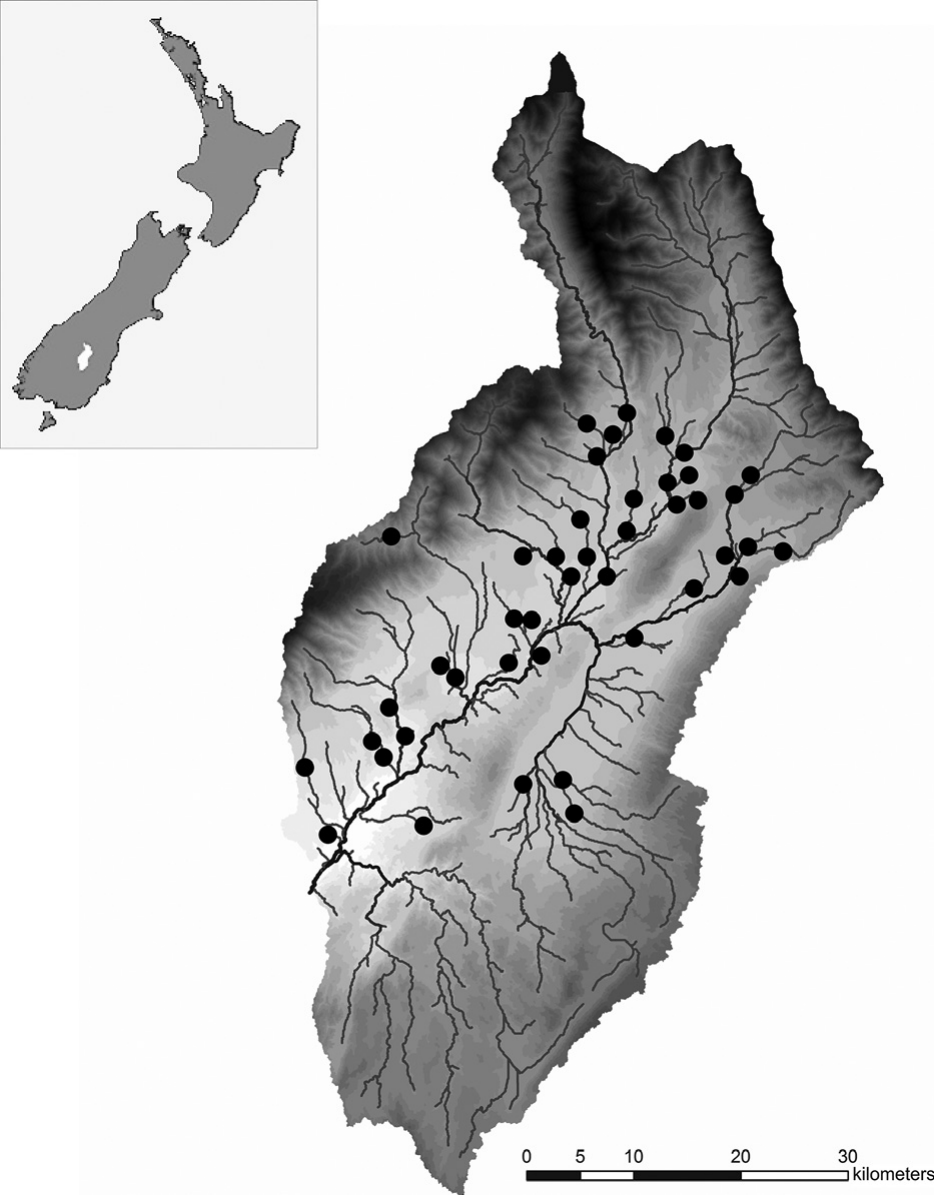
Location of the Manuherikia River catchment on the South Island, New Zealand (insert), and the 43 study sites in the catchment. Note that only streams of third order or higher are shown.

Our 43 stream sites covered the gradients of both % FI and % WA as evenly as possible (Fig. 1). Because several sites with high abstraction rates were located in areas of low FI and *vice versa* (often water is diverted into irrigation races and transported to other sub‐catchments), % FI and % WA of the sites were uncorrelated (*R* ^2^ = 0.005). The spatial distribution of the sites is shown in Fig. 2. Studied stream reaches ranged from third to fifth order and were mainly unshaded (five sites were partially shaded). All sites were sampled once in early autumn, between 21 March and 4 April 2011.

### Field sampling and sample processing

The concentration of total nitrogen in stream water and the amount of suspendable inorganic sediment deposited on the stream bed have been found to be the two reach‐scale variables most strongly related to our landscape‐scale stressors (among other nutrient and sediment variables: dissolved nitrate, dissolved phosphate, total phosphate, percentage covered with fine sediment and fine sediment depth), see Lange et al. (2014b). Consequently, we focus on the same two physicochemical variables in the present paper. At each site, total nitrogen was determined from three unfiltered water samples using standard methods (APHA 1998). The amount of suspendable inorganic sediment (particles ≤2 mm) was determined from five samples using the quantitative Quorer method (Clapcott et al. 2011).

We sampled benthic algae and cyanobacteria from the streambed surface by taking five samples at random within riffle habitats following standard quantitative methods (Biggs and Kilroy 2000). Samples were either scraped from rocks using a toothbrush or, at sites dominated by filamentous algae (15 of 43 sites), obtained by cutting a core from filamentous algal mats floating in the current. Both types of samples were collected inside a plastic cylinder (inner diameter 27 mm, height 25 mm) to standardize the surface area sampled. Samples were pooled for each site after collection and placed on ice in the field and frozen at −20°C until further analysis.

Sample preparation involved homogenization of the periphyton slurry with a blender (Omni Mixer; Ivan Sorval Inc., Newton, CT) for 30 sec and then preservation of a 15‐mL aliquot in 3% formalin for taxonomic identifications. We identified and enumerated at least 300 algal or cyanobacterial cells per sample at 400**×** magnification to the lowest practical taxonomic levels using an inverted microscope (Zeiss Axiovert 25, Jena, Germany) and standard keys (Prescott 1962; Cox 1996; Biggs and Kilroy 2000; Bellinger and Sigee 2011). For filamentous cyanobacteria with small cells, 10‐μm‐long units were counted and are referred to as “cells” henceforth.

### Trait database

We undertook an extensive literature search of biological traits for the algal and cyanobacterial species found in our samples and assigned 91 taxa to seven traits with 23 trait categories (Table 2; see Supplementary Information for details). Taxa were mostly classified according to their genus‐level identifications but species‐specific trait information was used where necessary (for cell size, life forms and resistance to disturbance). Table 2 includes the references used to assign the taxa to a specific trait category.

**Table 2:** The seven traits and their 23 categories (21 of which were included in the statistical analysis; see Methods)

Functional traits	Trait categories	Abbreviation for analysis
(1) Cell size Berthon et al. (2011), USGS (2002)	(1) nano (5 ≤ 100 μm^3^) (2) micro (100 ≤ 300 μm^3^) (3) meso (300 ≤ 600 μm^3^) (4) macro (600 ≤ 1500 μm^3^) (5) very large (>1500 μm^3^)	BIOVOLUME_c1 BIOVOLUME_c2 BIOVOLUME_c3 BIOVOLUME_c4 BIOVOLUME_c5
(2) Life form Burliga et al. (2004) Ferragut and de Campos Bicudo (2010)	(1) colonial (2) filamentous (3) flagellate (4) unicellular	LIFEFORM_colonial LIFEFORM_filamentous LIFEFORM_flagellate LIFEFORM_unicellular
(3) Attachment to substratum Biggs et al. (1998b)	(1) no fixation structure: entangled filaments, motile and filamentous diatoms; (2) attached: some filamentous algae, erect diatoms with pad/stalk (3) tightly attached: adnate and prostrate diatoms	ATTACHMENT_low ATTACHMENT_med ATTACHMENT_high
(4) Nitrogen fixation Stancheva et al. (2013)	(0) no (1) yes	NITROGENFIX_1
(5) Motility Round (1984)	(1) attached (2) gliding (3) drift	MOTILE_attached MOTILE_gliding MOTILE_drift
(6) Main reproductive technique Biggs et al. (1998b), Prescott (1962)	(0) fission (including binary fission in prokaryotes and mitosis in eukaryotes) (1) fragmentation (breaking of filaments)	REPRO_frag
(7) Spore formation Prescott (1962), Agrawal (2009) Souffreau et al. (2010)	(1) not forming spores (2) zoospores (no dormancy, asexual) (3) akinetes (thick cell wall, dormancy possible, asexual) (4) oospores and zygospores (thick cell wall, dormancy, sexual)	SPORES_none SPORES_zoos SPORES_akinetes SPORES_oos.zygs

### Data analysis

We investigated how the relative abundances of algal trait categories were associated with our landscape‐scale and reach‐scale predictor variables. For the traits nitrogen fixation and main reproductive technique, which were binary, only one of the two categories was included in the analysis, thus reducing the total number of trait categories from 23 to 21 (see Tables 2 and 3). First, we investigated how trait categories were related to the landscape‐scale variables % FI and % WA. Second, we used the reach‐scale variables nutrients and fine sediment (both ln‐transformed) to determine the best models in relation to these physicochemical predictors.

**Table 3:** Effect sizes (partial standardized regression estimates), 95% CIs and *R* ^2^ values of the final models for the relationships between traits and landscape‐scale predictor variables (for abbreviations see Table 2). Effect size categories (Nakagawa and Cuthill 2007): trivial <0.1, weak ≥0.1, moderate >0.3, strong >0.5). Only effect sizes ≥0.1 were considered to be biologically relevant, but predictor terms with coefficients <0.1 were kept in two cases where an interaction term was retained in the final model

Response	Transformation	FI^a^	FI × FI^b^	WA^c^	FI × WA^d^	*R* ^2^	Interaction
BIOVOLUME_c1	ln(*x*)	−0.33 (−0.62; −0.03)				0.10	
BIOVOLUME_c2	ln(*x*)	−0.45 (−0.96; 0.06)	0.74 (0.05; 1.42)	0.30 (−0.07; 0.67)	0.48 (−0.13; 1.08)	0.20	Antagonism
BIOVOLUME_c3	ln(*x*)	−0.23 (−0.54; 0.08)				0.05	
BIOVOLUME_c4	ln(*x*)						
BIOVOLUME_c5	ln(*x*)						
LIFEFORM_colonial	√						
LIFEFORM_filamentous		0.32 (0.02; 0.62)				0.10	
LIFEFORM_flagellate							
LIFEFORM_unicellular	ln(*x*)	−0.42 (−0.71; −0.13)		−0.01 (−0.28; 0.27)	0.27 (0.08; 1.18)	0.30	Antagonism
ATTACHMENT_high	ln(*x*)						
ATTACHMENT_low		0.29 (−0.01; 0.60)		−0.24 (−0.54; 0.06)		0.12	
ATTACHMENT_medium		−0.30 (−0.61; 0.00)		0.23 (−0.07; 0.53)		0.13	
NITROGENFIX_1	ln(*x*)	−0.61 (−1.00; −0.23)	0.61 (0.05; 1.17)			0.20	
MOTILE_attached		−0.46 (−0.74; −0.18)				0.21	
MOTILE_drift	ln(*x*)	0.33 (0.03; 0.63)				0.11	
MOTILE_gliding	ln(*x*)	−0.26 (−0.57; 0.05)		0.05 (−0.24; 0.34)	0.74 (0.16; 1.32)	0.22	Antagonism
REPRO_frag		0.31 (0.01; 0.61)				0.10	
SPORES_akinetes	ln(*x*)						
SPORES_none		−0.45 (−0.85; −0.05)	0.49 (−0.11; 1.1)	0.15 (−0.17; 0.47)	0.67 (0.1; 1.24)	0.29	Antagonism
SPORES_oos.zygs	√						
SPORES_zos	√	0.31 (0.01; 0.61)				0.09	

a % Farming intensity.

b Second‐order polynomial terms.

c % Water abstraction.

d Interaction terms.

We used general linear models and an information‐theoretic model‐selection approach, following Johnson and Omland (2004) (for details see Lange et al. 2014a). All analyses were run in R (version 3.0, R Development Core Team 2014). For the landscape‐scale analysis, the global model (intercept plus four predictor terms) comprised the first‐order terms % FI and % WA, the second‐order term FI × FI to describe unimodal relationships (as found in Lange et al. 2014a,b) and the interaction FI × WA. We also considered nested versions of the global model with one or more predictor terms removed, and the null model (intercept only) (resulting in eight competing models). The second‐order term WA × WA was not considered here because we had no hypotheses for non‐linear relationships with WA. For the reach‐scale analysis, we considered models with a maximum of five predictors: first‐order terms for fine SED (sediment) and NUT (nutrients), the second‐order terms SED × SED and NUT × NUT to describe unimodal relationships (as found in Lange et al. 2014a,b), and the interaction SED × NUT (resulting in 13 competing models).

Response and predictor variables were centred by subtracting the sample mean from each value and scaled with two standard deviations to allow the use of regression estimates as effect sizes (Schielzeth 2010). The full model sets were generated using the *dredge* function implemented in the *MuMIn* package (Barton 2013). If more than one top model was chosen, model averaging produced one final model for each response variable, with regression estimates and their 95% Confidence Intervals (CIs) calculated as weighted averages using Burnham and Anderson's (2002) “zero‐method”. Standardized partial regression coefficients represent effect size measures (categories: trivial <0.1, weak >0.1, moderate >0.3, strong >0.5) and their 95% CIs are measures of precision and uncertainty (Nakagawa and Cuthill 2007). We only considered effect sizes ≥0.1 as biologically relevant, but terms with coefficients <0.1 were kept in cases where the interaction term was retained in the final model. For comparison of stressor strengths, we calculated overall effect sizes and their respective standard errors for landscape and reach‐scale predictors from the variance‐weighted absolute values of the effect sizes in the final models (Nakagawa and Cuthill 2007).

## Results

**Figure 3:**
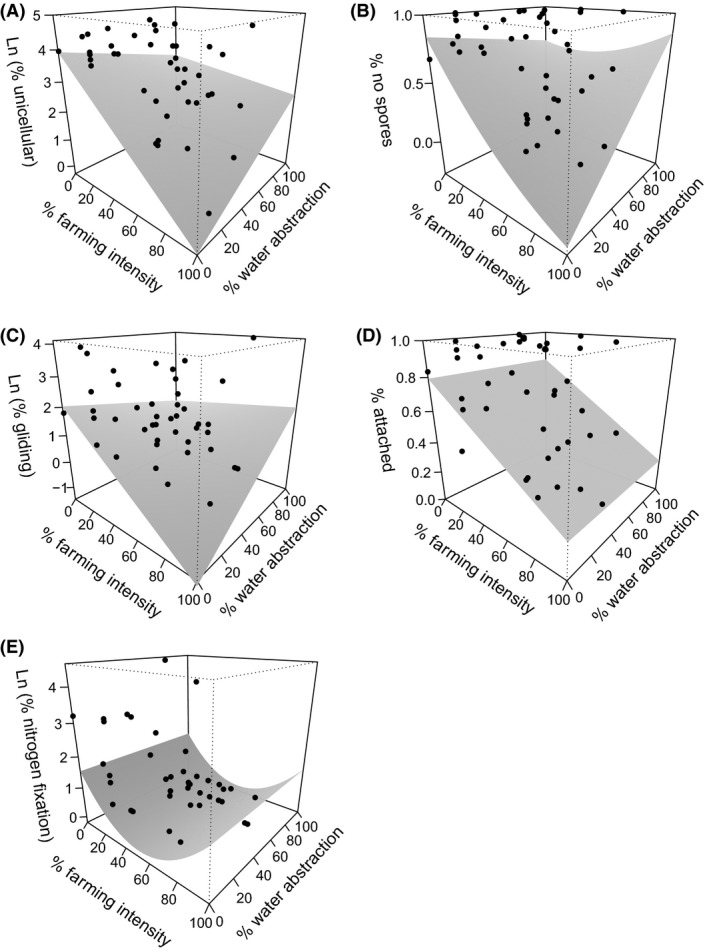
Examples of relationships between the trait categories (A) unicellular life form, (B) no spore formation, (C) gliding motility, (D) attached algae and (E) nitrogen fixation and the landscape‐scale predictor variables farming intensity and water abstraction (those with the highest *R* ^2^ values, see Table 3). The final models are shown with three‐dimensional response surfaces.

In the landscape‐scale analysis, 14 of the 21 investigated trait categories showed a relationship to % FI, six of these categories were also related to % WA, and four retained an interaction term (Table 3). All interactions were classified as antagonisms because the combined effects were smaller than expected based on the individual effects involved. The overall mean effect size (based on all individual linear or quadratic effects >0.10) of FI (0.40) was more than double that of WA (0.16). The strongest individual response pattern occurred for unicellular life forms, which showed a negative relationship to FI at low but not at high WA intensities (antagonistic interaction; *R* ^2^ 0.30) (Fig. 3). Next in strength came the relationships for cells lacking the ability to form spores and gliding cells (*R* ^2^ 0.29 and 0.22), best modelled as negative relationships with FI combined with overall positive relationships with WA and following an antagonistic response pattern. The patterns shown by attached algae and nitrogen‐fixing cells (*R* ^2^ 0.21 and 0.20) were all best modelled by a negative relationship with FI.

**Figure 4:**
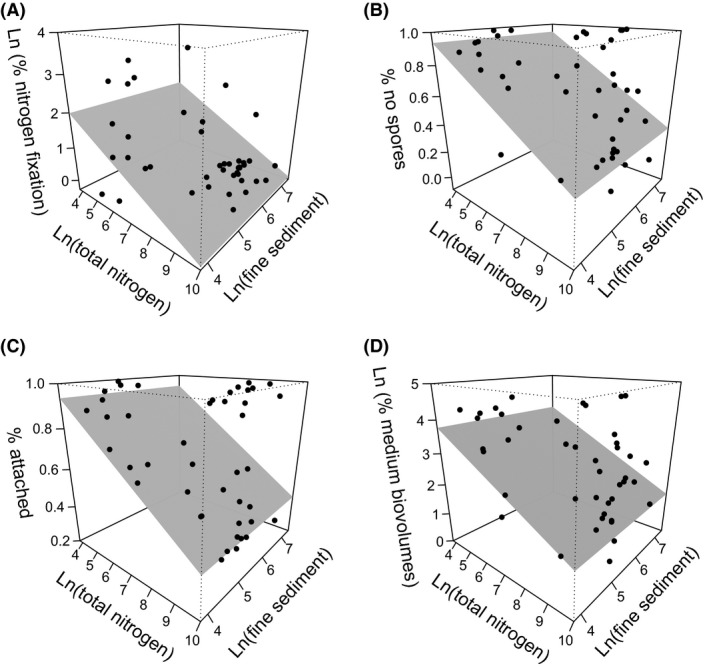
Examples of relationships between the trait categories (A) nitrogen fixation, (B) no spore formation, (C) attached algae and (D) medium biovolumes and the reach‐scale variables total nitrogen and fine sediment (those with the highest *R* ^2^ values, see Table 4). The final models are shown with three‐dimensional response surfaces.

In the reach‐scale analysis, 12 of the 21 trait categories included either nutrients (9) or fine sediment (3) in the final model (Table 4). The overall mean effect size of nutrients (0.34) was slightly larger than that of sediment (0.30). In relative terms, the strongest individual response patterns (in decreasing order) were for taxa with nitrogen‐fixing taxa, algae not forming spores, attached algae and medium biovolumes (with *R* ^2^ ranging from 0.19 to 0.10). All these were best modelled by medium‐sized negative relationships with total nitrogen concentrations (Fig. 4). Weak response patterns (*R* ^2^ 0.10–0.07) for gliding algae were best described by negative unimodal relationships with sediment, and formation of zoospores and large biovolume by positive relationships with sediment.

**Table 4:** Effect sizes (partial standardized regression estimates), 95% CIs and *R* ^2^ values of the final models for the relationships between traits and reach‐scale predictor variables (for abbreviations and other details see Table 2)

Response	Transformation	SED^a^	SED × SED^b^	NUT^c^	NUT × NUT^b^	SED × NUT^d^	*R* ^2^
BIOVOLUME_c1	ln(*x*)			−0.28 (−0.59; 0.02)			0.08
BIOVOLUME_c2	ln(*x*)						
BIOVOLUME_c3	ln(*x*)			−0.39 (−0.68; −0.10)			0.15
BIOVOLUME_c4	ln(*x*)	0.28 (−0.03; 0.58)					0.07
BIOVOLUME_c5	ln(*x*)						
LIFEFORM_colonial	ln(*x*)						
LIFEFORM_filamentous							
LIFEFORM_flagellate	√						
LIFEFORM_unicellular				−0.26 (−0.56; 0.05)			0.06
ATTACHMENT_high	ln(*x*)						
ATTACHMENT_low				0.29 (−0.01; 0.59)			0.08
ATTACHMENT_medium				−0.30 (−0.6; 0.00)			0.09
NITROGENFIX_1	ln(*x*)			−0.44 (−0.73; −0.16)			0.19
MOTILE_attached				−0.42 (−0.71; −0.13)			0.17
MOTILE_drift	ln(*x*)			0.28 (−0.02; 0.58)			0.08
MOTILE_gliding	ln(*x*)	−0.33 (−0.72; 0.07)	−0.55 (−1.21; 0.11)				0.10
REPRO_frag	ln(*x*)						
SPORES_akinetes	ln(*x*)						
SPORES_none				−0.44 (−0.72; −0.15)			0.19
SPORES_oos.zygs	√						
SPORES_zoos	√	0.29 (−0.01; 0.59)					0.08

a Ln‐transformed amount of suspendable fine sediment on the stream bed.

b Second‐order polynomial terms.

c Ln‐transformed total nitrogen concentrations in the stream water.

d Interaction terms.

## Discussion

### Mechanisms driving algal community composition

We tested our conceptual framework according to the ten specific hypotheses shown in Table 1. Our predictions were mostly supported by our findings (seven hypotheses supported, three rejected; individual hypotheses are discussed below). Moreover, in cases where hypotheses were not supported we gained new insights into processes governing algal communities in agricultural catchments. Farming intensity shaped communities by increasing nutrient supply, resulting in a community composition dominated by large, non‐attached, filamentous algae after several weeks of summer/autumn low‐flow conditions. Water abstraction, which we also consider to reduce the probability of flood‐induced periods of high shear stress, enhanced the severity of low flows, caused depositional conditions and increased the probability of stream drying, resulting in a community dominated by small, resilient and motile taxa at high levels of FI.

#### Morphological traits

Cell size is a key trait influencing growth rates, metabolism and access to the resources needed by benthic algae (Passy 2007b) and phytoplankton (Litchman and Klausmeier 2008; Kruk et al. 2010). Morphological traits showed frequent (and sometimes strong) relationships to our gradient of FI, whereas relationships with WA intensity were less pronounced. The proportion of small cell sizes decreased at higher levels of FI, implying that increased nutrient supply supported the growth of larger cell sizes with a smaller surface to volume ratio (supporting hypothesis MR1 [morphology and resources]).

Position within a three‐dimensional periphyton matrix determines access to nutrients and light, with improved access in taxa that can elevate themselves above others, for example by tall growth (Passy 2007a; Lange et al. 2011). Thus, filamentous algae were the dominant life form at high levels of FI in our study (supporting hypothesis MR2). A New Zealand‐wide study of 78 streams also reported that low flows and total nitrogen concentrations were the two key variables predicting the growth of filamentous algae (Snelder et al. 2014).

The same morphological traits may also be related to disturbance. In the context of our study of stream algae, we define disturbance broadly as any discrete event including severe low flows or stream drying, but also extreme fluctuations in temperature and oxygen conditions (all of which may arise due to intensive agriculture; Allan 2004) or grazing by herbivorous animals. Cell size is a key trait determining a species’ ability to recover after disturbance because smaller cells typically have higher growth rates that confer greater resilience (Passy 2007b; Litchman and Klausmeier 2008). We observed that small cells were favoured by increasing WA at high FI, presumably because they showed greater resilience to the higher risk of stream drying at increased WA intensities (supporting hypothesis MD1 [morphology and disturbance]).

Life form is important in relation to the ability to gather resources and also to withstand disturbance by high shear stress or grazing. We predicted in hypothesis MD2 that filamentous algae would increase at low shear stress (which should occur more often at high WA intensities). Instead we observed an increase in unicellular taxa with WA at high FI (rejecting hypothesis MD2). Unicellular organisms may have an advantage under depositional conditions causing increased sedimentation and maybe also during stream drying, due to their small size increasing the likelihood of entering crevices between substratum particles or shifting into deeper layers of the biofilm.

Directional flow is the most prominent feature of stream ecosystems and some algae have developed specific attachment mechanisms to maintain their position. Therefore we had predicted (hypothesis MD3) that attachment strength would be positively related to disturbance. However, flow reduction disfavoured non‐attached algae (while the proportion of algae closely but not tightly associated with the substratum increased), rejecting this hypothesis, and non‐attached (“floating”) forms dominated at higher levels of FI. Floating filamentous algae, often entangled within macrophytes, were a prominent feature of streams at high farming intensities and, at the reach‐scale, “floating” was positively related to nitrogen concentrations but not to fine sediment levels. Therefore “floating” also appears to be an advantageous strategy for gathering nutrients under stable flow conditions.

#### Physiological traits

Light and nutrients are essential resources for benthic algae and cyanobacteria. Light availability can be reduced by riparian vegetation, attenuation through the water column or in lower layers of the biofilm matrix (Hill 1996), but we did not quantify light levels in our study. Nitrogen fixation can provide advantages under nutrient‐limiting conditions (Stancheva et al. 2013). In our study, this key trait showed strong negative relationships to FI and stream water nitrogen concentrations but no relationships to WA or fine sediment (supporting our hypothesis PR1 [physiology and resources]). These findings suggest that FI exerted the strong observed effects on algal communities through an altered supply of nutrients.

#### Behavioural traits

We predicted an increase in actively motile algae with increasing resource supply but observed a decrease in attached algae and an increase in drifting organisms with FI (rejecting hypothesis BR1 [behaviour and resources]). Again, this implies that floating filamentous forms have an advantage in gathering nutrients under stable flow conditions. For motile algae, we found an increase with WA intensity (at high FI) and a unimodal relationship (an initial increase followed by a decrease) with deposited fine sediment (supporting hypothesis BD1 [behaviour and disturbance]). Paralleling these findings, motile algal taxa were also more common downstream of sites with WA in a survey of 10 Hong Kong streams (Tang et al. 2013), and similar positive relationships for this trait with fine sediment were reported in stream channel experiments manipulating sediment cover and depth (Piggott et al. 2012, 2015). The latter patterns may suggest that increased habitat heterogeneity afforded by low levels of fine sediment inputs provides suitable habitat for species that move on or through the sediment and live within crevices (Schneck et al. 2011), but that this initial subsidy is followed by a decline of these growth forms at high sediment levels.

#### Life‐history traits

Vegetative reproduction and dispersal by fragmentation increased at higher levels of FI but showed no relationship with WA intensity (rejecting hypothesis LHD1 [life‐history and disturbance]). Overall, dispersal by fragmentation is slower than dispersal by fission (binary fission in prokaryotes and mitosis in eukaryotes) because of larger propagule sizes (Blinn et al. 1998). Reproduction by fragmentation is typical of filamentous forms that have an advantage under resource‐rich conditions. Spore formation may provide such taxa that reproduce by fragmentation with the ability to tolerate unfavourable conditions instead of relying on recolonization from undisturbed habitats.

The proportion of taxa unable to form spores (zoospores, akinetes, oospores or zygospores) showed an antagonistic response pattern in our survey, with an overall negative relationship to FI and a positive relationship to WA. The cell structures of akinetes, oospores and zygospores in comparison to vegetative cells give them the advantage of being able to tolerate unfavourable conditions and remain viable for long periods (Agrawal 2009). Spore formation may therefore increase resilience of large‐celled filamentous algae at high farming intensities since these organisms usually also have limited dispersal abilities (De Bie et al. 2012). Because high FI often increases disturbance frequency and/or intensity (Allan 2004), the positive relationship of the prevalence of spore‐forming algae with FI supports hypothesis LHD2. To our knowledge, spore formation has not been investigated together with other traits such as cell size and growth forms but could be an important trait to consider in the future. Further, spore formation might be analogous to resistance forms (Dolédec et al. 2006) or diapausing or otherwise protected egg stages (Williams 1996), traits often investigated in stream invertebrates in conjunction with environmental stress and community resilience.

### Which traits showed the strongest relationships to environmental gradients?

Morphological, physiological, behavioural and life‐history traits all showed similarly strong relationships to our gradients of anthropogenic stressors at both the landscape and the reach scale. In agreement with this overall result of our study, diatom size classes, growth forms and life forms (mobile, colonial, tube‐forming, stalked and pioneer) all provided a similar degree of discrimination along a gradient of organic pollution in a survey of 328 sites from 212 rivers in South‐West France (Berthon et al. 2011). In the past, growth‐form traits were often the only ones considered to detect algal or diatom responses to environmental conditions in stream surveys or reach‐scale field experiments (for diatoms only: Pringle 1990; Passy 2007a; Berthon et al. 2011; Stenger‐Kovács et al. 2013; Goldenberg Vilar et al. 2014; including all algae: Schneck et al. 2011; Schneck and Melo 2012) or mesocosm experiments (for diatoms only: Lange et al. 2011; Passy & Larson 2011; Rimet and Bouchez 2011; including all algae: Piggott et al. 2012; Magbanua et al. 2013; Wagenhoff et al. 2013; Piggott et al. 2015). However, our study did not investigate the responses of algal growth forms because we consider growth forms to constitute guilds and not single traits. Growth form integrates several traits such as life form, attachment mechanism and motility, which can make it difficult to assign taxa to a particular guild. Thus, classification of taxa into growth forms often relies on expert knowledge, and this contradicts the fundamental principle that traits should be easily measurable (Poff 1997; Weithoff 2003; Kruk et al. 2010). Consequently, we recommend that future trait‐based assessments of algal communities should encompass detailed morphological, physiological, behavioural and life‐history traits, rather than growth forms, to gain a better understanding of the mechanistic drivers of community composition.

In our survey, nitrogen fixation and spore formation showed stronger relationships to environmental gradients (higher *R* ^2^ values and effect sizes) than did morphological traits at the reach scale. Note that diatoms cannot form spores (Souffreau et al. 2010) and few diatom species can host nitrogen‐fixing endosymbionts (*Epithemia* and *Rhopalidoa*) (Bellinger and Sigee 2011). Therefore, we recommend that assessments of stream periphyton in future trait‐based studies should include cyanobacteria and other algal groups, not just diatoms. We concur with Kelly (2013) that researchers should move away from “admiring empty diatom frustules” and extend their scope to include soft‐bodied algae. Paralleling our findings, nitrogen fixation was also the trait that provided the best differentiation along a gradient of nutrient enrichment in field experiments in Californian streams (Nelson et al. 2012). Traits such as nitrogen fixation and pigment composition, which relate to resource availability, may therefore turn out to be important in detecting alterations to energy and matter fluxes caused by increasing nitrogen deposition and climate change.

We excluded several algal traits from our conceptual framework. Maximum linear dimension and surface to volume ratio (Law et al. 2014) were excluded because we were unable to gather this information from the literature; both traits, however, are strongly related to cell biovolume which was readily available (USGS 2002). Other traits proposed for ecological assessment include saprobic and trophic indices, and tolerances to pollution, salinity and pH (Kelly et al. 2008; Porter 2008; Schmidt‐Kloiber and Hering 2011). Note that these are all defined by relating trait abundance to environmental variables and therefore do not conform with the concept that biological traits should be easily measurable without reference to the environment (Poff 1997; Weithoff 2003; Kruk et al. 2010).

The relatively large amount of unexplained variation for algal trait variables in our observational study compared to some others (particularly tightly controlled manipulative experiments) may be due to sampling across a wide gradient of land‐use intensities and not including in our analysis additional factors that influence periphyton communities (see also Nelson et al. 2012; Algarte et al. 2014). Such factors include water temperature (Piggott et al. 2015), invertebrate grazing pressure (Steinman 1996) and pesticide concentrations (Rimet and Bouchez 2011), which are all rarely measured in stream surveys.

### Can algal traits help disentangle multiple‐stressor effects?

In our landscape‐scale analysis, algal traits were unable to distinguish between the gradients of farming and WA intensity. All final models performing better than the null model included FI as a predictor and none of the traits solely showed a relationship to WA. Our combined findings imply that FI had more pronounced and pervasive effects on stream algae than WA, though WA did modulate algal responses to FI. Farming activities (e.g. application of fertilizer and manure, stock density, stock access to waterways) determine, among other variables, the amount of nutrients and fine sediment reaching agricultural streams. Water abstraction, by reducing stream flows and creating depositional conditions, can then enhance sedimentation and organic matter processing. Paralleling our findings, nutrient regime but not flood frequency had an effect on algal taxon richness in a 15‐month study of 12 New Zealand streams (Biggs and Smith 2002), and algal community composition was more affected by nutrient supply than by hydrology in three New Zealand rivers (Biggs et al. 1998a). Moreover, our results for algal traits are similar to those for invertebrate trait distributions in the Manuherikia River catchment (Lange et al. 2014b), where FI also had more severe effects than WA.

At the stream reach scale, relationships of algal traits with nutrient concentrations were far more frequent and stronger than for fine sediment, but their comparable overall effect sizes imply that both physicochemical variables are important. This finding suggests that algal communities in the Manuherikia catchment are more responsive to changes in nutrient supply than to changes in substratum characteristics. In contrast to the landscape‐scale analysis, at the reach scale we only identified single‐stressor relationships and no interactions among stressors. A stream mesocosm experiment examining algal communities and three growth forms along wide gradients of nutrients and fine sediment also reported that interactions between these two stressors were either weak or non‐significant (Wagenhoff et al. 2013). By contrast, another mesocosm study investigating the effects of nutrient and fine sediment on algal communities and five growth forms along a water temperature gradient found frequent interactions between all three stressors (Piggott et al. 2015). However, both these experiments focused on algal community composition and growth forms (instead of life forms) and considered only one or two algal “traits” (see related discussion above). These contrasting findings imply that (1) we should investigate non‐additive responses of algal communities with a comprehensive set of traits, and (2) algal traits may be suitable for disentangling the effects of multiple stressors at the reach scale (e.g. nitrogen fixation to detect nutrient enrichment and motility to detect fine sediment inputs).

## Conclusions and outlook

Our conceptual framework based on a fairly comprehensive set of biological traits of benthic algae and cyanobacteria allowed us to gain insights into the mechanisms by which FI and WA affect stream algal communities. Farming intensity, the more pervasive catchment‐scale stressor in our survey, acted mainly through increased inputs of nutrients, disadvantaging small, nitrogen‐fixing taxa while favouring large, non‐attached filamentous algae with limited dispersal abilities. There is increasing evidence that fine sediment may be the “master stressor” for stream invertebrate and fish communities (see reviews by Jones et al. 2011; Kemp et al. 2011). In our survey on stream algae, by contrast, nutrient concentrations were more important than fine sediment levels in the reach‐scale stressor analysis. To our knowledge, our study is the first to investigate interactive effects of hydrological alteration (flow reduction and increased intermittency) and eutrophication on freshwater periphyton, even though both stressors are among the five main threats for freshwater biodiversity (Dudgeon et al. 2006). This knowledge gap, and also the interplay of these stressors with stressors arising from global change (Woodward et al. 2010; Piggott et al. 2015), should be addressed by future research.

More generally, we encourage researchers to employ our trait‐based approach in field surveys and manipulative experiments to gain a better understanding of the mechanisms by which resource supply, disturbances and/or substratum characteristics shape stream algal communities. The key challenge to be overcome before this approach can be applied widely is the availability of detailed species‐specific trait information. The development of trait databases for freshwater algal communities has begun (Porter 2008; Schmidt‐Kloiber and Hering 2011), but these need to be extended to include a more comprehensive list of traits.

Future research in this area could apply our conceptual framework to other river catchments and experimental settings and refine the framework by (1) assessing the roles of ecological trade‐offs among traits, phylogenetic constraints and the so‐called trait syndrome (correlations among traits and intraspecific or interspecific trait co‐variance) (Poff et al. 2006; Menezes et al. 2010; Verberk et al. 2013; Laughlin and Messier 2015), (2) including additional traits (e.g. surface to volume ratio, pigmentation, ability to produce mucilage) once enough information becomes available in the literature, and (3) considering continuous traits as opposed to categorical traits (also dependent on data availability).

## Conflict of Interest

The authors declare no conflict of interest.

## Supporting information


**Table S1.** List of the 91 periphyton taxa found in our samples and their assignment to the 7 biological traits with 23 trait categories.
